# Laparoscopic right-sided traumatic diaphragmatic hernia repair: a case report and video vignette

**DOI:** 10.1093/jscr/rjaf862

**Published:** 2025-10-31

**Authors:** Charlotte Cornwell, William A Ziaziaris, Bryan M Tran, Jerome M Laurence

**Affiliations:** Department of Upper Gastrointestinal Surgery, Royal Prince Alfred Hospital, 50 Missendon Road, Camperdown, NSW 2050, Australia; Department of Upper Gastrointestinal Surgery, Royal Prince Alfred Hospital, 50 Missendon Road, Camperdown, NSW 2050, Australia; Department of Upper Gastrointestinal Surgery, Royal Prince Alfred Hospital, 50 Missendon Road, Camperdown, NSW 2050, Australia; Department of Upper Gastrointestinal Surgery, Royal Prince Alfred Hospital, 50 Missendon Road, Camperdown, NSW 2050, Australia

**Keywords:** traumatic diaphragmatic hernia, laparoscopic surgery

## Abstract

Traumatic diaphragmatic hernias (TDHs) are uncommon and frequently missed, with right-sided defects particularly rare due to hepatic protection. Delayed presentations may result in complications including obstruction or strangulation. We present the case of a 70-year-old female with a right-sided TDH with incarceration of small bowel, 7 years following blunt trauma. Laparoscopic repair was undertaken in left lateral position, requiring mobilization of the right liver to access the posterolateral defect. Incarcerated small bowel was reduced and found to be viable. The diaphragmatic defect was closed in two layers using non-absorbable V-Loc™ sutures. The patient recovered uneventfully and was discharged on postoperative day four. This case highlights the value of laparoscopy for both diagnosis and repair, and the technical considerations required for right-sided access. Primary closure is preferred for small defects, while mesh reinforcement may be necessary in larger or chronic cases to reduce recurrence.

## Introduction

Traumatic diaphragmatic hernias (TDHs) are rare complications of thoracoabdominal trauma, with right-sided hernias particularly uncommon due to the protective effect of the liver. These injuries are often missed, leading to delayed presentations resulting in significant morbidity [1]. Herniation typically occurs through a defect in the posterolateral diaphragm, allowing abdominal contents to migrate into the thoracic cavity, causing obstruction, strangulation, and/or respiratory compromise. Laparoscopy has become an increasingly valuable tool for both the diagnosis and repair of diaphragmatic injuries, even in delayed cases [2–6]. In this case report, we present the laparoscopic management of a delayed right-sided traumatic diaphragmatic hernia, illustrating the diagnostic challenges, surgical approach, and technical nuances involved in treating this rare pathology.

## Case report

**Figure 1:**
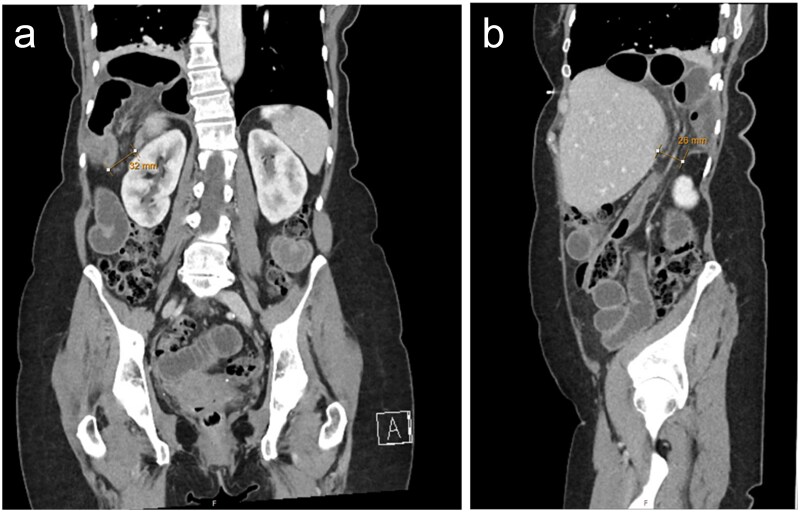
CT with a) coronal and b) sagittal slices demonstrating right sided posterolateral diaphragmatic defect containing incarcerated small bowel.

A 70-year-old female presented to the Emergency Department at our quaternary hospital with a 2-day history of abdominal pain, vomiting and obstipation. Her background was significant for hypertension and an appendicectomy at the age of 6. Seven years prior, the patient sustained right sided rib fractures following a fall. A computed tomography (CT) scan of the chest, abdomen and pelvis demonstrated a large right-sided diaphragmatic hernia containing incarcerated small bowel with reduced mucosal enhancement concerning for evolving ischaemia (Fig. 1). A diagnosis of delayed traumatic diaphragmatic hernia was made, and the patient was booked and consented for emergent laparoscopy.

The patient was placed in the left lateral position and optical entry performed through the right rectus muscle. Three working ports were placed in the epigastrium and right subcostal region, with the primary operator and assistant standing on the patient’s left side. Adhesions in the right upper quadrant were taken down and the right hemi-liver was mobilized, incising the right triangular ligament with hook diathermy. Once mobilized, the assistant’s right-hand port was used for anteromedial retraction of the liver. The hernia was identified and the hernial orifice slightly extended with diathermy to facilitate safe reduction of the incarcerated bowel. The viable small bowel contents were reduced and inspected. The diaphragmatic defect was then repaired in two layers using a non-absorbable 2–0 V-Loc™ suture.

The patient’s post-operative course was uneventful. She was commenced on full fluids and then progressed to a normal diet on post-operative day-1 and 2,respectively, and was discharged on post-operative day-4. At 6-week post-operative follow-up the patient was clinically well and a progress CT demonstrated no evidence of hernia recurrence.

## Discussion

Delayed TDHs, particularly on the right side, present significant diagnostic and surgical challenges due to their subtle initial presentation, the protective effect of the liver, and the risk of progressive herniation of intra-abdominal contents [7, 8]. Once diagnosed, timely surgical repair is essential to prevent complications including obstruction, strangulation, or respiratory compromise [1, 9]. In our case, the patient presented with an incarcerated hernia, highlighting the potential for delayed TDHs to progress silently until they become acutely symptomatic. Laparoscopic repair has become an increasingly preferred approach, offering diagnostic clarity, improved surgical ergonomics in the left and right upper quadrants and reduced morbidity from the minimally invasive approach [2, 5]. However, optimal repair technique, specifically whether to perform primary suture closure or use mesh reinforcement, remains a topic of debate, particularly in delayed presentations where chronic defects may be large or fibrotic.

Optimal surgical exposure is critical. Patient positioning typically involves a slight reverse Trendelenburg tilt, utilizing gravity to displace abdominal organs inferomedially to enhance visualization of the defect. Mobilization of the liver is often required to access the defect, which requires division of the right triangular ligament to allow medial retraction of the liver. The assistant plays a vital role in maintaining exposure by dynamically retracting the liver and diaphragm, providing the primary surgeon with a clear operative field to reduce herniated contents and perform the repair. Effective teamwork during this step is essential to minimize intraoperative complications and ensure precise mesh placement or suture repair. These technical considerations help overcome the unique challenges posed by the right-sided location and contribute significantly to the success of a laparoscopic approach.

Primary closure with non-absorbable sutures, ideally tension-free and often in two layers, is widely recommended for small defects [9]. Use of absorbable sutures and repair under tension increases risk of recurrence [10]. Potential complications from primary suture closure include tension-related suture failure, re-injury from diaphragmatic motion, and negative intrathoracic pressure, all of which may contribute to hernia recurrence [3, 11].

Mesh is advised when defects are too large (>3 cm) for primary closure without tension and may reduce recurrence [9]. Biological, biosynthetic or composite meshes may be used and can be fixated using tackers or transfascial sutures [4, 6]. Meticulous fixation of the mesh, including adequate overlap of at least 3-5 cm and secure anchoring, is essential to prevent recurrence caused by mesh displacement or failure at the prosthesis - host interface [12]. Mesh may provoke erosion, adhesion or and infection, and composite or biologic meshes are preferred to limit bowel adhesion [13, 14].

Delineating operative strategy based on defect size, hernia chronicity, tissue quality, and contamination is critical. If ischaemic bowel or dense adhesions are encountered, conversion to laparotomy may be required [15]. For large or chronic defects, mesh reinforcement offers improved tensile integrity and lower recurrence risk. In smaller defects without tension, primary non-absorbable sutures suffice and avoid mesh-related risks.

## Conclusion

Laparoscopic repair of delayed right-sided TDHs is both safe and effective when performed with careful patient selection and surgical planning. Primary suture repair remains the technique of choice for small defects, provided a tension-free closure can be achieved. In contrast, larger or chronic defects may benefit from mesh reinforcement. The choice of mesh should be individualized, balancing the need for strength and durability with the risk of complications such as adhesion, erosion, or infection. Meticulous surgical technique, including secure fixation, adequate mesh overlap, and careful handling of the hernia contents is essential to minimize recurrence and postoperative complications. This case report illustrates the feasibility and safety of minimally invasive repair for delayed traumatic diaphragmatic hernia, highlighting key operative strategies including patient positioning, exposure techniques, and methods of defect closure.

## Supplementary Material

Laparoscopic_Right-Sided_Traumatic_Diaphragmatic_Hernia_Repair_rjaf862 Video Vignette displaying the operative technique used to repair a right-sided traumatic diaphragmatic hernia.
